# Sourdough Fermentation of Wheat Flour does not Prevent the Interaction of Transglutaminase 2 with α_2_-Gliadin or Gluten

**DOI:** 10.3390/nu7042134

**Published:** 2015-03-25

**Authors:** Niklas Engström, Ann-Sofie Sandberg, Nathalie Scheers

**Affiliations:** Division of Food and Nutrition Science, Department of Biology and Biological Engineering, Chalmers University of Technology, S-412 96 Gothenburg, Sweden; E-Mails: ann-sofie.sandberg@chalmers.se (A.-S.S.); nathalie.scheers@chalmers.se (N.S.)

**Keywords:** celiac disease, gluten intolerance, lactic fermentation, sourdough, G12 antibody, tissue transglutaminase, TG2, QLP, α_2_-gliadin

## Abstract

The enzyme transglutaminase 2 (TG2) plays a crucial role in the initiation of celiac disease by catalyzing the deamidation of gluten peptides. In susceptible individuals, the deamidated peptides initiate an immune response leading to celiac disease. Several studies have addressed lactic fermentation plus addition of enzymes as a means to degrade gluten in order to prevent adverse response in celiacs. Processing for complete gluten degradation is often harsh and is not likely to yield products that are of comparable characteristics as their gluten-containing counterparts. We are concerned that incomplete degradation of gluten may have adverse effects because it leads to more available TG2-binding sites on gluten peptides. Therefore, we have investigated how lactic acid fermentation affects the potential binding of TG2 to gluten protein in wheat flour by means of estimating TG2-mediated transamidation in addition to measuring the available TG2-binding motif QLP, in α_2_-gliadin. We show that lactic fermentation of wheat flour, as slurry or as part of sourdough bread, did not decrease the TG2-mediated transamidation, in the presence of a primary amine, to an efficient level (73%–102% of unfermented flour). Nor did the lactic fermentation decrease the available TG2 binding motif QLP in α_2_-gliadin to a sufficient extent in sourdough bread (73%–122% of unfermented control) to be useful for celiac safe food.

## 1. Introduction

### 1.1. Celiac Disease

Celiac disease (CD) is a disease with an autoimmune component in tissue transglutaminase, which is initiated by the ingestion of gluten proteins in wheat and related proteins in barley (hordeins) and rye (secalins) in genetically predisposed individuals. Exposure will lead to various degrees of inflammation and damage to the small intestine [1]. The majority of patients will experience an improvement in their physical and psychological condition after starting on a gluten-free diet, which to date is the only treatment available [2]. The main genetic risk factors for developing CD are the genotypes encoding for the HLA class II molecules HLA-DQ2 and HLA-DQ8, where the majority of patients carry the DQ2 heterodimer [3,4]. However, roughly one third of the population in Europe carries the DQ2 heterodimer, which means that there are other factors contributing to disease development [5]. Other factors thought to increase the risk for developing CD are various types of infections and the age of gluten introduction [6,7,8,9]. CD can be diagnosed at any age but it is most commonly discovered at early childhood, following the introduction of gluten in the diet, and at around the age of 40 and 50 for women and men, respectively [10,11].

### 1.2. Deamidation of Gluten Initiates Celiac Disease

The enzyme transglutaminase 2, also referred to as tissue transglutaminase (TG2, EC 2.3.2.13), plays a crucial role in the initiation of CD [9,12,13]. As reviewed by Park *et al.* (2010), TG2 is expressed ubiquitously and can be found in, e.g. the cytoplasm, the nucleus, on the extracellular cell surface as well as in the extracellular matrix [14]. It is present in the mucosa of the small intestine and more highly expressed in untreated CD patients [9,15]. TG2 is a member of a family of Ca^2+^-dependent enzymes that catalyze transamidation or deamidation reactions of, e.g. gluten peptides. In the first step of the enzymatic reaction, the γ-carboxamide of a peptide-bound glutamine acts as an acyl donor that binds to a cysteine at the active site of TG2, resulting in the formation of a γ-glutamylthioester bond and the release of ammonia. In the second part of the reaction, the enzyme is deacylated either by aminolysis or hydrolysis. In the deacylation reaction, a primary amine, such as peptide-bound lysine, acts as acyl acceptor forming an amide bond (transamidation). Transamidated gluten does not result in a celiac response. In the hydrolysis reaction, water acts as acyl acceptor leading to the deamidation of glutamine into glutamic acid [16,17,18]. The deamidated gluten has a much higher affinity for the HLA-DQ2 and DQ8 heterodimers on antigen-presenting cells [9,12,13]. Once the deamidated gluten peptides are presented to CD4^+^ T-cells they produce pro-inflammatory cytokines and the subsequent inflammation leads to the degradation of the extracellular matrix and apoptosis of enterocytes leading to the celiac lesion [19]. CD4^+^ T-cells also activate B-cells, which differentiate into plasma cells that produce antibodies against both gluten and TG2 [19].

### 1.3. Potential Use of Sourdough in Bread Making to Decrease the Celiac Response

Several studies address the possibility of sourdough fermentation as a means to reduce the level of gluten proteins in bread [20,21,22,23,24,25]. However, to accomplish this, stringent methods are often used that are sometimes impractical or unrealistic to be able to yield products that are of comparable characteristics, quality and price as their gluten-containing counterparts. Full degradation of gluten requires long fermentations with a combination of several strains of different lactic acid bacteria and/or with the addition of a suitable peptidase. By fully degrading wheat gluten, the viscoelastic properties are lost, which reduces the benefit of the process. Incomplete degradation may lead to the retention of part of the beneficial properties but we are concerned that the protein breakdown may lead to more available TG2-binding sites on gluten peptides and thus increase deamidation reactions by TG2, which would increase the intra-tissue accumulation of antigen and thus potentiate the immune response in celiacs.

Therefore, we have investigated the effect of lactic acid fermentation on potential binding of TG2 to gluten proteins in wheat flour and breads made under similar conditions as industrially baked sourdough breads available on the consumer market. We used two methods: (1) a commercial antibody-based assay that recognizes the TG2-binding motif QLP present in α_2_-gliadin, considered to be the most immunogenic peptide derived from gluten proteins during digestion. The assay was used to indicate how many QLP motives remain available for TG2 binding after lactic fermentation and *in vitro* digestion; (2) a transamidation assay that indicates the interaction of TG2 with gluten proteins as an estimate of the interaction of *any* binding site, independent of the identity of gluten proteins. A primary amine is added in the assay to favor transamidation instead of deamidation reactions, which are undetectable in the present conditions. The experiments were made with three lactic acid bacterial strains (*L. plantarum*, *L. pentosus* and *L. brevis*) independently.

## 2. Materials and Methods

### 2.1. Sample Preparation

#### 2.1.1. Strains and Culture Conditions

Three *lactobacillus* subspecies (*L. brevis*, *L. plantarum*, and *L. pentosus*) were cultivated in M.R.S. broth (Oxoid Ltd., Hampshire, UK; 37 °C, 150 rpm). Overnight cultures inoculated at OD_600_ = 0.5 were prepared for the wheat flour fermentations (American Biosciences Ultrospec 10 cell density meter, Blauvelt, NY, USA). Colony forming units (CFU; Tryptone glucose extract agar, Sharlau SL, Barcelona, Spain; 24–48 h, 30 °C) were done to estimate bacteria concentration and to check for contamination. The cultures were washed by centrifugation (5 min, 1000 g) and re-suspended in NaCl-solution (0.9%) followed by a second centrifugation (5 min, 1000 g) and re-suspension in NaCl-solution (0.9%).

#### 2.1.2. Lactic Fermentation of Wheat Flour

Wheat flour (extraction rate 80%; Saltå Kvarn, Järna, Sweden) was fermented using the three different lactic acid bacteria. Flour (5 g) was mixed with water (MQ; 70 mL) and respective strain in NaCl-solution (0.9%, 5 M) resulting in a final OD_600_ of approximately 0.5. The flour slurries were allowed to ferment during shaking (21 h, 37 °C, 150–200 rpm). The samples were immediately frozen (−20 °C). The control was made in the same way but without the fermentation step.

#### 2.1.3. Sourdough Bread Making

Each *Lactobacillus* strain was transferred, in NaCl-solution (0.9%, 5 mL), to a mixture of equal amounts (*w*/*w*) of wheat flour and tap water, which was let to ferment 24 h in ambient temperature (sourdough). The sourdough (175 g) was mixed with fresh flour (350 g), tap water (200 g), commercial baker’s yeast (12.5 g; Kronjäst, Rotebro, Sweden), olive oil (10 g; Zeta Originale, Stockholm, Sweden) and NaCl (7.5 g; Santa Maria Bordssalt, Mölndal, Sweden). The sourdough constituted 20% of the total flour used for the bread. All the ingredients were mixed and kneaded in a KitchenAid (10 min, speed 2; Benton Harbor, MI, USA). The dough was leavened for 2 h in the bowl and was then divided into two parts which each were manually kneaded for approximately 1 min before left to rise for another 2 h. The breads were put on a hot baking plate and baked until reaching satisfactory crust coloration (18–25 min, approx. 220 °C). After baking the breads were freeze-dried (Heto DW6-55 Drywinner, Allerød, Denmark) and ground.

#### 2.1.4. *In Vitro* Digestion of Wheat Flour and Sourdough Bread

In order to simulate the impact of the gastric and proximal duodenal digestion, an *in vitro* digestion procedure using pepsin was conducted for all samples. The reasoning for terminating the digestion at early duodenal phase is that the digesta may come in contact with TG2 before the secretion of pancreatic enzymes. The fermented wheat flour samples (5 g) were mixed with water (MQ; 50 mL). The freeze-dried sourdough bread samples (0.34 g) were mixed with water (MQ; 54.76 mL) and NaCl-solution (0.9%, 0.34 mL), to correspond to the water content in the slurry of the fermented wheat flour. The simulated gastric digestion was started by lowering the pH to approximately 2.1 with HCl (5 M) and adding pepsin (porcine pepsin, Sigma Aldrich, St. Louis, MO, USA) dissolved in HCl (0.1 M, 1.5 mL, 406400 U/mL) and incubated (1 h, 37 °C, 150 rpm). The digestion was terminated by raising the pH to approximately 7 with NaOH (0.5 M). The samples were stored at −20 °C until further analysis. All samples were centrifuged (15 min, 5000 g) before assay analysis.

### 2.2. Detection of the TG2 Binding Motif QLP in α_2_-Gliadin

An antibody-based assay was done according to the instructions provided with the kit (GlutenTox ELISA Competitive; Biomedal Diagnostics, Sevilla, Spain), with a few modifications. The sample extraction was done at room temperature during 5 minutes with constant mild agitation, after which the samples were centrifuged (5 min, 2500 g). The sample dilution was 1:250. In brief, the samples were incubated with the G12-HRP conjugated antibody, which specifically recognizes the amino acid sequence QPQLPY that is present in α_2_-gliadin [26]. The sample-antibody mixture was added to a 96-well plate coated with gliadin, to capture residual antibodies. After washing, the gliadin-bound G12-HRP was allowed to react with the substrate, before adding H_2_SO_4_ (1 M) and measuring the absorbance at 450 nm.

### 2.3. TG2-Mediated Transamidation Assay

The transamidation assay was done according to Skovbjerg *et al*. [27] with a few modifications. Sample proteins were coated on 96 well plates onto which TG2 incorporated 5-(biotinamido)pentylamine; Eu-labeled streptavidin was subsequently added to bind to biotin; after which the chelated europium was measured by time-resolved fluorescence (345 nm excitation; 617 nm emission; Safire2; Tecan Group Ltd.; Männedorf; Switzerland). Tris-HCl (5 mM, pH 7.5; Gibco, Life Technologies, Stockholm, Sweden) and bovine serum albumin (BSA; 1%; Pierce/Thermo Scientific, Life Technologies, Stockholm, Sweden) were used as negative controls.

### 2.4. Statistics

For the fermented wheat flour, four separate fermentations were made for each strain and the control. For the sourdough bread, three breads were prepared for each strain and the control. When detecting the QLP binding motif, two replicates were made for each trial (*n* = 8–12 measurements). In the transamidation assay five to eight replicates were made for each trial (*n* = 27–32 measurements)). Two-tailed student’s *t*-test was used for significance testing (IBM SPSS Statistics 22). Differences were considered to be significant at *p* < 0.05.

## 3. Results

### 3.1. The Fermentation Process

The lactic fermented wheat flour samples showed no visible growth of other microorganisms as observed by examination of streaked TGE-agar plates. The starting pH of the controls was 6.3 and 6.0–6.2 for the samples. At the end of the fermentations (24 h), the pH was 3.2–3.8. Approximately 4 × 10^7^–9 × 10^8^ cells/mL dough was added to each sourdough as estimated by the CFU count. After *in vitro* digestion, all samples were between pH 6.9–7.2.

### 3.2. Fermentation with L. plantarum Significantly Increases Available QLP Motives on α_2_-Gliadin

**Figure 1:**
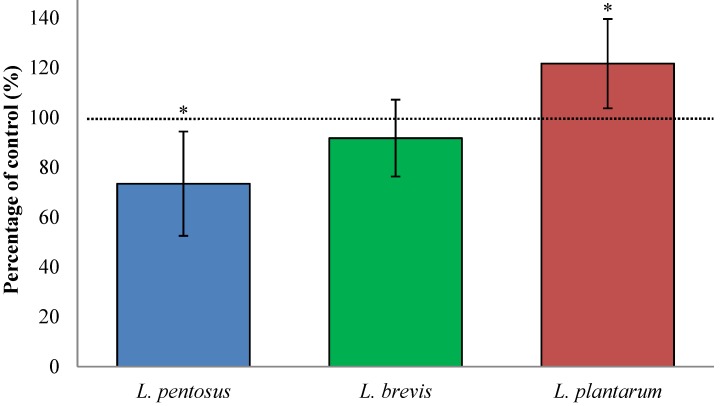
This bar graph shows the change in number of the TG2 binding motif QLP, specific to α_2_-gliadin, which remains available for TG2 binding after lactic fermentation and *in vitro* digestion of wheat flour. Wheat flour fermented with *L. brevis*, *L. pentosus*, or *L. plantarum* was compared to unfermented breads (control = 100%). Data are presented as means ± SD (*n* = 8–12) and shows the change 73%–122% of control. The asterisk indicates a significant change (*p* < 0.05) from control.

Lactic fermentation of wheat flour with *L. plantarum* significantly increased available QLP sites on α_2_-gliadin (22% ± 18%, *p* = 0.047, Figure 1) while the other *lactobacilli* subspecies reduced the binding motif either significantly (*L. pentosus*; 27% ± 21%, *p* = 0.027) or insignificantly (*L. brevis*; 8% ± 15%, *p* = 0.317) compared to unfermented wheat flour. Since α_2_-gliadin is considered to be the most immunogenic peptide in gluten, fermentation of wheat flour with *L. plantarum* may thus potentiate the toxicity of gluten to celiacs. Although, *L. pentosus* decreased the available TG2 binding sites on α_2_-gliadin, the extent (27% ± 21%) is not sufficient to be of any importance in the aim to use lactic fermentation as a means to detoxify gluten in wheat flour.

### 3.3. Fermentation with *L. plantarum* Slightly Decreased TG2-Mediated Transamidation of Gluten

**Figure 2:**
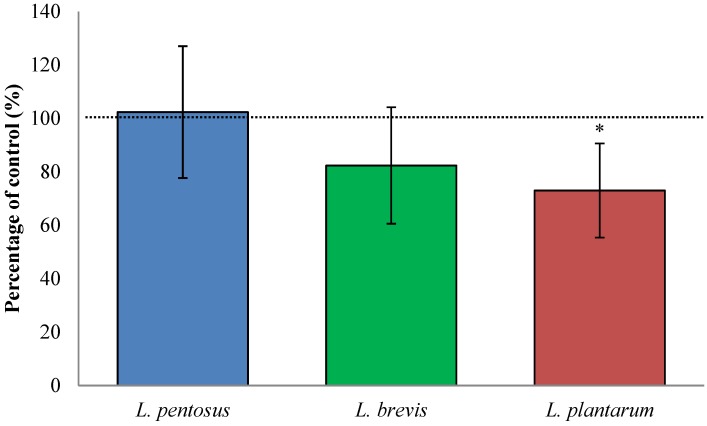
TG2-mediated transamidation of *in vitro* digested, fermented and unfermented, wheat flour in the presence of a primary amine indicates the interaction of TG2 and various binding sites on gluten protein. The change in transamidation caused by lactic fermentation was 73%–102% of the control. Data are presented as means ± SD (*n* = 27–32). The asterisk indicates a significant change (*p* < 0.05) from control.

By measuring the TG2-mediated transamidation, we get a measure of TG2 interaction with all gluten peptides in the flour sample, as in opposition to the antibody based assay, in which we focus on the interaction of TG2 with α_2_-gliadin (33 amino acid-long peptide, often referred to as the 33-mer). Fermentation of wheat flour with *L. plantarum* significantly decreased the transamidation of gluten (27% ± 18%, *p* < 0.001, Figure 2), while fermentation with *L. brevis and L. pentosus* did not affect the transamidation significantly (decrease of 18% ± 22%, *p* = 0.073 and increase of 2% ± 25%, *p* = 0.564, respectively). Important to remember is that despite that *L. plantarum* decreased, the total interaction of TG2 with gluten, the available binding sites on the most immunogenic peptide in gluten, was significantly increased.

### 3.4. Sourdough Fermentation with L. plantarum Decreased Available QLP Motives in α_2_-Gliadin

**Figure 3:**
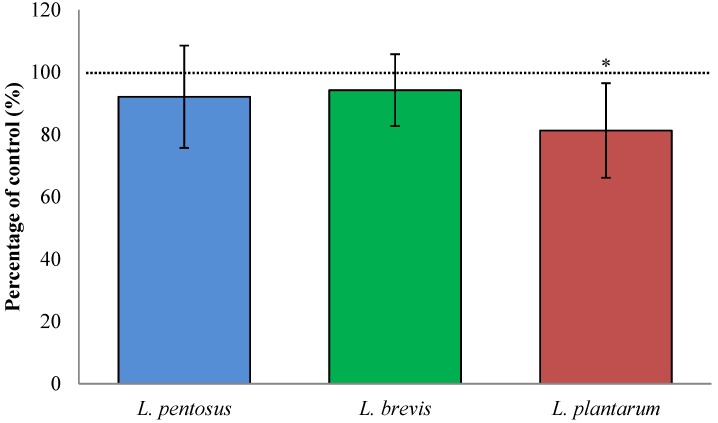
The change in the number of TG2 binding motif QLP in α_2_-gliadin from *in vitro* digested sourdough breads. The sourdough was fermented with *L. brevis*, *L. pentosus*, or *L. plantarum*. Changes were compared to those from unfermented control breads (control = 100%). Data are presented as means ± SD (*n* = 8–12). The asterisk indicates a significant change (*p* < 0.05) from control. Data show changes in the range 81%–94% of control.

In digested sourdough bread in which the sourdough part (20%) was fermented with *L. plantarum*, the available TG2 binding sites were decreased (19% ± 15%, *p* = 0.042; Figure 3) compared to control breads, leavened with baker’s yeast only. Sourdough fermented with *L. pentosus* and *L. brevis* did not significantly decrease available TG2 binding sites (8% ± 16%, *p* = 0.359 and 6% ± 12%, *p* = 0.390, respectively). The *L. plantarum* fermented wheat flour was fermented for 24 h while the *L. plantarum* fermented sourdough was fermented for 4 h (20% of total bread weight was fermented for 24 h), which suggests that a longer fermentation time produces more celiac toxic α_2_-gliadin peptides, than a shorter time.

## 4. Discussion

### 4.1. Is It Beneficial to Degrade Gluten?

Several studies have shown that fermentation with multiple selected strains of lactic acid bacteria and/or the use of specific enzymes may successfully degrade gluten [20,21,22,23,28,29]. These studies do not address if the processing affects the TG2 binding or the formation of deamidation products by TG2. Total degradation of wheat gluten would eliminate the viscoelastic properties, which are responsible for, e.g., the leavening capacity of bread or other baked products. Bread baked of wheat flour with no gluten will be perceived as inferior compared to a gluten-containing wheat bread. Wheat flour rendered gluten free by lactic fermentation and/or enzymatic treatment may, however, be used together with naturally gluten free flours and structuring agents to make products of similar quality as gluten-containing counterparts [21,22,25]. Full degradation of gluten requires long fermentation times (~24 h), often in combination of several strains of different lactic acid bacteria and with the addition of peptidases. Long fermentation times and addition of enzymes also mean higher production costs for the final product. Verification of the degradation is important, since a partial degradation of gluten may lead to an increased number of available TG2-binding sites as we observed in the present study. The increased interaction of TG2 with gluten or gluten-derived peptides may increase the load of deamidation products in the gastrointestinal tissues and thus potentiate or initiate the immune response in celiacs.

### 4.2. Evaluation of Methods for TG2 and Gluten Interactions

We have evaluated the use of different methods for estimating the interactions of TG2 with gluten, such as measuring the level of (1) deamidation and transamidation, (2) the ammonia release upon binding of TG2 to gluten, and (3) the specific recognition of an antibody to TG2 binding motives. After evaluation and optimization, we dismissed two of the methods due to poor reproducibility, namely the ammonia release and deamidation measurements. The ammonia release predicts the total binding of TG2 to gluten since ammonia is released upon the formation of γ-glutamylthioester bonds during a SN_2_ substitution reaction between TG2 and glutamine side chains in gluten. This gives us the total binding of TG2 to gluten, regardless of the subsequent transamidation or deamidation reaction. Which of these reactions will follow depends on the availability of primary amines. If no such amine is present, TG2 uses water instead to deamidate the substrate [19]. However, due to the low levels and volatile nature of ammonia, it proved difficult to obtain reliable results with this method. We also analyzed the samples for the deamidation product glutamate using a commercial deamidation assay (Glutamate Colorimetric Assay Kit, BioVision, San Francisco, CA, USA). The enzyme mix supplied with the assay uses glutamate as a substrate leading to color development, which can be measured spectrophotometrically. However, this assay proved not to be suitable for our samples.

## 5. Conclusions

We conclude that lactic fermentation of wheat flour does not sufficiently prevent TG2 interaction with gluten or decrease available TG2 binding sites on α_2_-gliadin, compared to unfermented flour. Prolonged fermentation times with *L. plantarum* (24 h) even increased available TG2 binding sites on α_2_-gliadin, indicating that lactic fermentation may not be an appropriate method for making celiac safe products.
